# Both mature miR-17-5p and passenger strand miR-17-3p target TIMP3 and induce prostate tumor growth and invasion

**DOI:** 10.1093/nar/gkt680

**Published:** 2013-08-28

**Authors:** Xiangling Yang, William W. Du, Haoran Li, Fengqiong Liu, Anna Khorshidi, Zina Jeyapalan Rutnam, Burton B. Yang

**Affiliations:** ^1^Sunnybrook Research Institute, Sunnybrook Health Sciences Centre, Toronto, M4N 3M5, Canada and ^2^Department of Laboratory Medicine and Pathobiology, University of Toronto, Toronto, M5S 1A8, Canada

## Abstract

MicroRNAs (miRNA) precursor (pre-miRNA) molecules can be processed to release a miRNA/miRNA* duplex. In the canonical model of miRNA biogenesis, one strand of the duplex is thought to be the biologically active miRNA, whereas the other strand is thought to be inactive and degraded as a carrier or passenger strand called miRNA* (miRNA star). However, recent studies have revealed that miRNA* strands frequently play roles in the regulatory networks of miRNA target molecules. Our recent study indicated that miR-17 transgenic mice could abundantly express both the mature miR-17-5p and the passenger strand miR-17-3p. Here, we showed that miR-17 enhanced prostate tumor growth and invasion by increasing tumor cell proliferation, colony formation, cell survival and invasion. miRNA target analysis showed that both miR-17-5p and miR-17-3p repressed TIMP metallopeptidase inhibitor 3 (TIMP3) expression. Silencing with small interfering RNA against TIMP3 promoted cell survival and invasion. Ectopic expression of TIMP3 decreased cell invasion and cell survival. Our results demonstrated that mature miRNA can function coordinately with its passenger strand, enhancing the repressive ability of a miRNA by binding the same target. Within an intricate regulatory network, this may be among the mechanisms by which miRNA can augment their regulatory capacity.

## INTRODUCTION

Prostate cancer is the most common malignant tumor in men, representing ∼29% of diagnoses every year in the USA. As an increasing number of men aged <50 years are diagnosed with prostate cancer (1), the development of sensitive diagnostic tools and treatments will be key to treating this disease. MicroRNAs (miRNAs) have been shown strong potential as diagnostic, prognostic and therapeutic biomolecules in prostate cancer owing to their ability to function as tumor suppressors or oncogenic reagents. More than 50 miRNAs have been reported as being deregulated in prostate cancer (2). For instance, miR-221/222 has been found to be involved in the development and metastasis of prostate cancer (3). miR-21 is another potential oncogenic miRNA overexpressed in prostate cancer and acts as a key regulator contributing to tumor growth, invasiveness and metastasis (3). miR-125b is upregulated in clinical samples and affects prostate cancer tumorigenesis and androgen independency by targeting EIF4EBP Bak1 (4). In addition, miR-20a, which is part of the mir-17–92 cluster, is overexpressed in prostate cancer, and its inhibition induces cell death and apoptosis in PC3 cells (5).

MiRNAs are short strands of RNAs of 18–24 nt in length (6). Most miRNAs bind and target the 3′-untranslated region (3′UTR) of mRNAs with imperfect complementarity and function as translational repressors, which have implication in cancer development (7,8). MiRNA processing is a complex mechanism that involves ribonuclease III type enzymes, which cleaves long double-stranded RNA molecules (9,10) in the nucleus. Nuclear precursor RNAs are cleaved by the endonuclease Drosha to release precursor miRNAs, which are 60–70 nt long imperfect hairpin structures (10–13). After being transported to the cytoplasm, the precursor miRNAs are processed by the endonuclease Dicer, producing a mature miRNA and a passenger strand. The mature miRNA is the guide strand for regulation of gene expression (14), whereas the passenger strand is believed to be degraded and inactivated (15,16). Following the nomenclature, miRNA precursors that generate two kinds of abundant miRNAs by instance such mature sequences are denoted the miR-#-5p (5′ arm) and miR-#-3p (3′ arm). However, accumulating evidence has suggested that miRNA star strands (miRNA*) can be loaded into Ago2 protein and contribute to regulating mRNA translation (17–21).

Previously, we founded that transgenic mice expressing the miR-17 precursor produced comparable levels of mature miR-17-5p and passenger miR-17-3p (22). Our results suggested that both strands may play roles in gene regulation. In this report, we studied the role of miR-17 in prostate cancer *in vivo* and *in vitro*. We found that both miR-17-5p and miR-17-3p could target TIMP3 and coordinately function as an oncogene in prostate cancer.

## MATERIALS AND METHODS

### Construct generation

The pre-miR-17 was ligated into a mammalian expression vector, BluGFP, which contains a Bluescript backbone, a CMV promoter driving expression of green fluorescent protein and a H1 promoter driving miR-17 as described previously (22).

A luciferase reporter vector (pMir-Report; Ambion) was used to generate the luciferase constructs. A fragment of the 3′UTR of TIMP3 with the potential binding site for miR-17-5p was cloned using two primers, hsa-TIMP3-SacI (5′ttcgagctctgagcgccagaccctgccccacctca) and hsa-TIMP3-MluI-R17-5p (5′ccacgcgtgacctttctttaaatgttccaagtgc) by PCR. The PCR products were digested with SacI and MluI, and the digested fragment was inserted into a SacI- and MluI-digested pMir-Report luciferase plasmid, to obtain a luciferase construct Luc-TIMP3-R17-5p. A mutant construct was generated with two primers hsa-TIMP3-SacI and hsa-TIMP3-MluI-R17-5p-mut (5′ccacgcgtgacctttctttaaatgttccttcagctaaat) using similar approach.

A fragment of the 3′UTR of TIMP3 with the potential binding site for miR-17-3p was cloned using two primers, hsa-TIMP3-R17*-SacI (5′g**gagctc**ggaacctgtattcctcttcttcgt) and hsa-TIMP3-R17*-MluI (5′cc**acgcgt**gacagacacagtctggttgggaca) by PCR. The PCR products were digested with SacI and MluI, and the digested fragment was inserted into a SacI- and MluI-digested pMir-Report luciferase plasmid, to obtain a luciferase construct Luc-TIMP3-R17-3p. A mutant construct was generated with two primers hsa-TIMP3-SacI and hsa-TIMP3-R17*-MluI-mut (5′cc**acgcgt**gacagacacagtctggttgggacaacgtgtgg) using similar approach.

To study the function of TIMP3 in miR-17-regulated cell activities, we generated TIMP3 expression construct. The coding sequence of TIMP3 cDNA was subcloned using two primers huTimp3-kozak-BamHI (5′cccggatccgccgccaccatgaccccttggctcgggctcatc) and huTimp3-XbaI (5′ctatctagatcaggggtctgtggcattgatgatgc) in a PCR. The PCR product was digested with BamHI and XbaI and inserted into the pcDNA3.1 vector (Invitrogen, Hygromycin resistant).

### Real-time PCR

Total RNAs were extracted from cell cultures with mirVana miRNA Isolation Kit (Ambion) according to the manufacturer’s instructions. RT-PCRs were performed as previously described. For mature miRNA analysis, the total RNAs were extracted from 1 × 10^6^ cells, followed by first strand cDNA synthesis using 1 µg of RNA. In the following steps, a PCR was performed with QuantiMir-RT Kit using 1 µl of cDNA as template. To perform these experiments, other kits were also needed including miScript Reverse Transcription Kit, cat#218060, miScript Primer Assay, cat#218411 and miScriptSYBR GreenPCR Kit, cat#218073 from Qiagen. The primers specific for mature miR-17-5p and miR-17-3p were purchased from Qiagen. The primers used as real-time PCR controls were human-U6RNAf (5′gtgctcgcttcggcagcacatatac) and Human-U6RNAr (5′aaaaatatggaacgcttcacgaatttg).

### Cell proliferation assay

Cells (1 × 10^5^ cells per well for DU145; 5 × 10^4^ cells per well for PC3 and LNCaP cells) were seeded on 6-well tissue culture dishes in Dulbecco’s modified Eagle’s medium (DMEM) containing 5% fetal bovine serum (FBS). Cells harvested were counted on Day 2, 4, 7 or as indicated.

For cell-cycle analysis, cells were seeded on 6-well plates at 40% confluence and incubated at 37°C for 24 h. The cells were harvested, washed with ice-cold phosphate buffered saline and resuspended in ice-cold 70% ethanol for 30 min. The cells were then treated with 10 µg/ml RNase at 37°C for 30 min. The cells were spin down and stained with 10 µg/ml PI for 30 min. The DNA content was measured by flow cytometry with FlowJo software (FACS Calibur; Becton Dickinson, CA).

### Tumorigenic assay in nude mice

Five-week-old strain CD1 nude mice were injected with the miR-17- or vector-transfected cells at the cell number of 5 × 10^5^ cells or indicated in the legends in 50 µl of phosphate buffered saline with 50 µl of Matrigel per mouse. Tumors sizes were measured using a caliper by measuring the length (L) and width (W) of the tumors for volume calculation (tumor volume V = (L × W^2^)/2). After 14 days, the mice were sacrificed, and the tumors were removed. Tumors were fixed in 10% buffered formalin (Histochoice Tissue Fixative MB, Amresco), processed and embedded in paraffin. Immunohistochemistry was performed on 4 µm of paraffin sections mounted on charged slides. The sections were stained with hematoxylin and eosin (H&E).

### Immunohistochemistry

Organs were freshly fixed in 10% neutral buffered formalin overnight, immersed in 70% ethanol and finally embedded in paraffin, followed by microtome section (Leica RM2255). Sections (4 µm of thickness) were deparaffinizated in two changes of xylene for 5 min each and rehydrated by placing three times in 100% ethanol for 3 min each. Endogenous peroxidase activity was blocked by incubating the sections in 3% H_2_O_2_ solution in methanol at 4°C for 20 min and washed twice in TBS for 5 min each. Antigen retrieval to unmask antigenic epitope was performed by heating the sections in sodium citrate buffer (pH 6.0) in a microwave presser cooker for 4 min. Non-specific reaction with cellular proteins was blocked with 10% normal goat serum at room temperature for 30 min. The slides were then incubated in a humidified chamber at 4°C overnight with primary antibodies against TIMP3 (1:500, Abcam), PTEN (1:500, Millipore) and p21 (1:500, BD) in TBS containing 10% normal goat serum and 1% bovine serum albumin. The sections were washed three times in TBS for 5 min each and incubated with secondary antibody solution at 37°C for 45 min. The slides were then processed with ABC (Vector labs) in the same conditions and stained with Diaminobenzidine (DAB) according to manufacturer’s protocols. The slides were subsequently stained with Mayer’s Hematoxylin for counter staining followed by slide mounting.

### Western blotting

Cell lysates were prepared from cells cultured in 6-well tissue culture plates by lysing the cells in each well with 100 μl of lysis buffer containing protease inhibitors. Protein concentrations were measured by Bio-Rad protein assay kit. Lysates containing 30–100 μg of protein were subject to SDS–PAGE. The separated proteins were transferred to a nitrocellulose membrane followed by immunostaining with a primary antibody at 4°C overnight. Next day, the membrane was washed and incubated with HRP-conjugated goat-anti-mouse secondary antibody at room temperature for 2 h followed by ECL detection. After detection of protein bands, the blot was re-probed with anti-GAPDH antibody to confirm equal loading of samples.

### Cell survival assay

Cells (1.5 × 10^5^ or 5 × 10^5^ cells per well) were seeded on 35-mm tissue culture dishes in DMEM containing 10% FBS. The medium was changed to serum-free medium 24 h after the inoculation and incubated for different periods. The cell numbers were counted by using trypan blue staining as described (20,23).

### Colony formation in soft agarose gel

Colony formation was assessed using a method previously described (24). In brief, 10^3^ cells were mixed in 0.3% low-melting agarose (Seaplaque, FMC) in DMEM supplemented with 10% FBS and plated on 0.66% agarose-coated 6-well tissue culture plates, which prevented attachment of cells to the plate surface. Three weeks after cell inoculation, colonies were fixed with cold methanol and stained with Coomassie brilliant blue for examination of colonies.

### Cell invasion

Cells (1 × 10^4^ per well for DU145 and PC3 cells; 2 × 10^4^ per well for LNCaP cells) suspended in serum-free medium were loaded into transwell membrane inserts that were pre-coated with 100 µl of Matrigel that had been diluted (1:10-fold). The inserts were placed in 24-well plates, which contained medium supplemented with 10% FBS. Cells were incubated at 37 °C and allowed to migrate and invade through the Matrigel and the membrane pores in the inserts. The upper Matrigel layer and cells were removed 48 h after cell inoculation. The cells on the surface of the lower side of the membrane were fixed with 100% methanol and stained with Coomassie brilliant blue. Cells that migrated onto the lower surface were counted from representative areas for quantification.

### Small interfering RNA assay

DU145 cells were seeded at the density of 2 × 10^5^ cells per well in 6-well plates in 2 ml of culture medium containing 10% FBS. The cells were transfected with small interfering RNAs (siRNAs) by using Lipofectamine™ 2000 (Invitrogen). The cells were harvested 48 h after the transfection and then subjected to various assays.

### Luciferase activity assay

Luciferase activity assay was performed using a dual-luciferase reporter system developed by Promega (E1960) as described previously (25). In brief, DU145 cells were seeded onto 24-well tissue culture plates at a density of 3 × 10^4^ cells/well in the medium containing 10% FBS and cultured for 24 h. The cells were co-transfected with the luciferase reporter constructs, corresponding miRNA mimics and Renilla luciferase construct by Lipofectamine 2000. The cells were then lysed using 100 μl of passive lysis buffer per well on a shaker for 30 min, and the lysates were centrifuged for supernatant collection. Twenty microliters of the supernatants were then mixed with 100 μl of LAR II, and the firefly luciferase activities were measured using a luminometer. For the internal control, 100 μl of Stop & Go reagent was added to the samples. Renilla luciferase activities were then measured in the same tubes. Luciferase activities between different treatments were compared after normalization with Renilla luciferase activities. The miRNA concentrations used were 100 nM.

### Statistical analysis

The results (mean values ± SE) of all the experiments were subject to statistical analysis by *t*-test. The levels of significance were set at *P < 0.05 and **P < 0.001.

## RESULTS

### miR-17 promotes tumor cell proliferation, survival and colony formation

**Figure 1.**
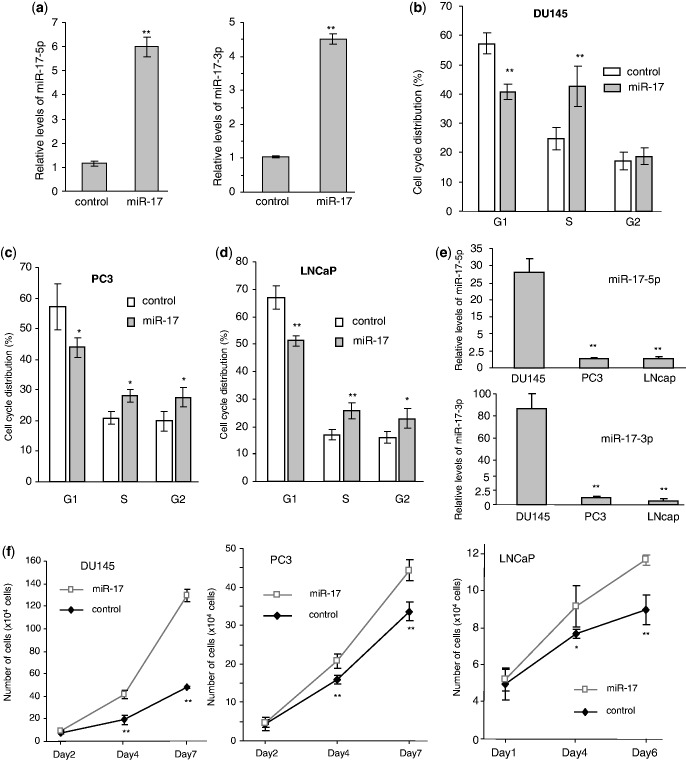
miR-17 increased cell-cycle progression and cell proliferation. (**a**) Mature miR-17-5p and miR-17-3p levels were measured by real-time PCR in the *miR-17*- and vector-transfected DU145 cells. U6 acted as an internal loading control. Asterisks indicate significant differences. **P < 0.01. Error bars indicate SD (*n* = 3). (**b**) Cell cycle progression was measured by flow cytometry. Expression of miR-17 decreased the number of cells in the G1 phase. **P < 0.01, *n* = 3. (**c** and **d**) Analysis of cell cycle progression indicated that expression of miR-17 decreased the number of cells in the G1 phase in PC3 cells (c) and LNCaP cells (d). (**e**) Expression of miR-17-5p (upper) and miR-17-3p (lower) was measured by real-time PCR in Du145, PC3 and LNCaP cells. Only DU145 cells expressed high levels of both miRNAs. (**f**) Cell proliferation was assayed in Du145, PC3 and LNCaP cells. Cells transfected with miR-17 displayed increased proliferation compared with the control. Error bars, SD (*n* = 3), **P = 0.02.

**Figure 2.**
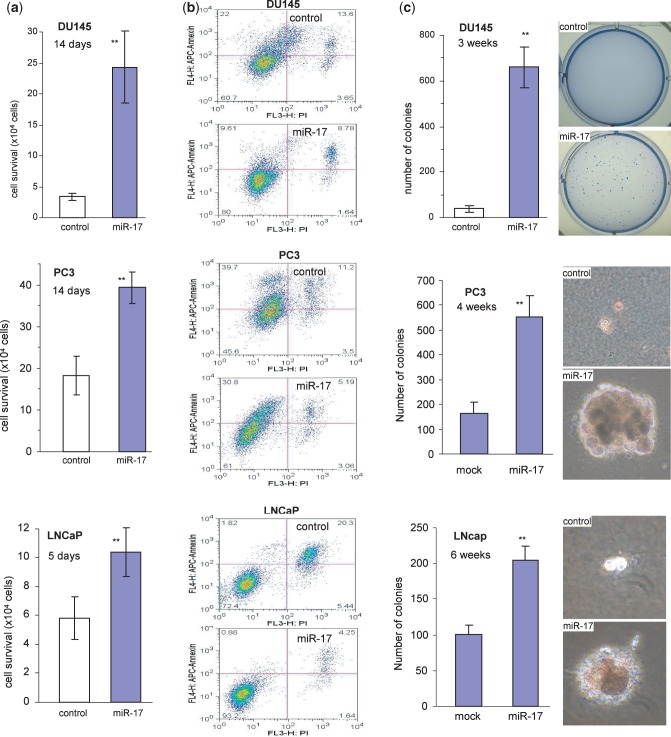
miR-17 promotes cell survival and colony formation but decreased apoptosis. (**a**) DU145 (top), PC3 (middle) and LNCaP (bottom) cells transfected with miR-17 or a control vector were maintained in tissue culture dishes in serum-free conditions for cell survival assay. Transfection with miR-17 enhanced cell survival. **P < 0.001, *n = *8. (**b**) Apoptosis of the miR-17- and control vector-transfected DU145, PC3 and LNCaP cells cultured in serum-free medium for 2 days was measured. More apoptotic cells, Annexin V positive, were detected in the vector-transfected cells than in the miR-17-transfected cells. (**c**) The miR-17-transfected DU145, PC3 and LNCaP cells formed more and larger colonies per plate than the control when the cells were plated in low melting agarose with 10% FBS.

Previous studies demonstrated that miR-17 played roles in the growth of a number of cancer types (26–29). We examined whether miR-17 played a role in prostate cancer cell activities. We stably transfected human prostate cancer cell lines DU145 with our previously generated miR-17 expression construct (22) and confirmed that the levels of miR-17-5p and miR-17-3p expressions were significantly higher in the transfected cells than the control cells (Figure 1a). The effect of miR-17 on cell cycle progression was examined, and we found that miR-17 transfected DU145 cells had significantly less cells detected in the G1 phase than the control cells (Figure 1b). Two other prostate cancer cell lines PC3 and LNCaP were also stably transfected with miR-17 and the control vector. Cell cycle analysis indicated that expression of miR-17 decreased G1 populations in both PC3 (Figure 1c) and LNCaP (Figure 1d) cells. Analysis of endogenous miRNA showed that DU145 cells expressed significantly higher levels of miR-17-5p and miR-17-3p, as compared to PC3 and LNCaP cells (Figure 1e). Cell number was quantified at 2, 4 and 7 days after inoculation to determine proliferation rates. In all cell lines, the miR-17 cells showed a significantly higher proliferative capacity as compared with the control (Figure 1f). Cell survival was examined in serum-free conditions. Expression of miR-17 in DU145, PC3 and LNCaP cells displayed enhanced cell survival compared with the control cells (Figure 2a). Measurement of cell apoptosis indicated that the miR-17-transfected cells underwent apoptosis at a lower rate than the control cells (Figure 2b). The miR-17- and vector-transfected cells were also cultured in soft agarose. Cells transfected with miR-17 formed more and larger colonies than those transfected with the control vector (Figure 2c).

### miR-17 enhances tumorigenesis and invasion

**Figure 3.**
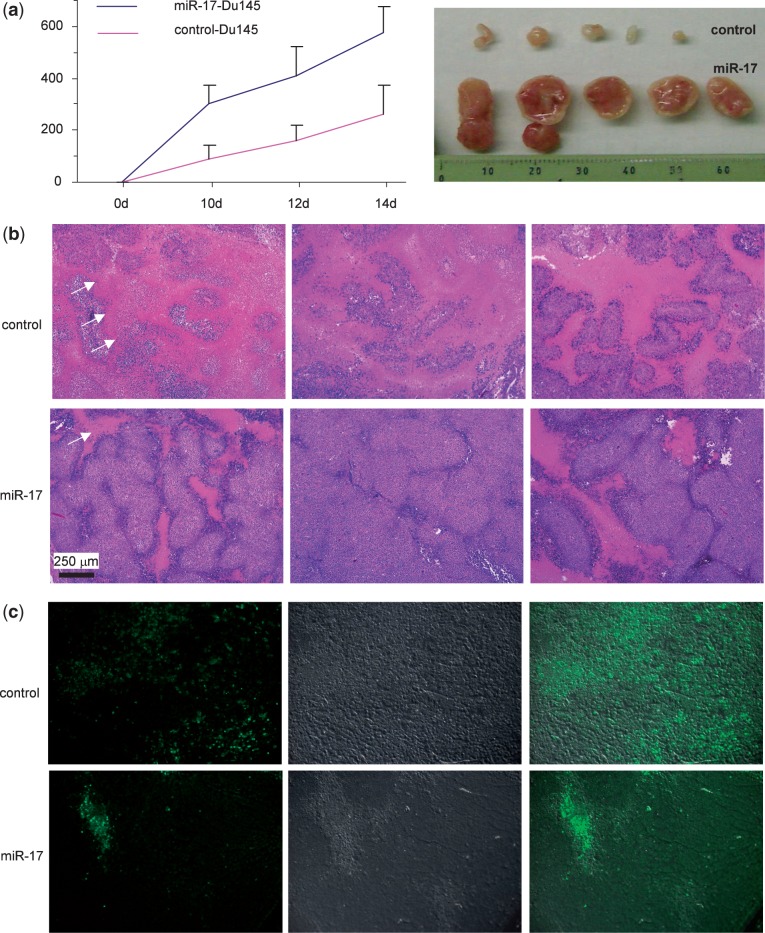
miR-17 enhances tumor growth in DU145 cells. (**a**) DU145 cells transfected with miR-17 or the control vector were injected subcutaneously into nude mice. Tumor growth was monitored for 14 days. Expression of miR-17 increased tumor growth. Typical sizes of tumors are shown (right). (**b**) The H&E stained tumor sections were examined under a light microscope. Evidence of extensive cell death (pink) could be seen in the control but not in the miR-17 tumors. (**c**) Tumor sections were subject to TUNEL staining (green). The miR-17 tumor cells had fewer apoptotic cells than the control.

We tested whether miR-17 played a role in tumor growth. CD-1 nude mice were subcutaneously injected with DU145 cells transfected with miR-17 or the control vector. Expression of miR-17 produced an increased rate of tumor growth as compared to the control cells (Figure 3a, left). In an extreme case, cells transfected with miR-17 formed tumors with >10-fold than the control cells (Figure 3a, right). These results suggested that the miR-17-transfected cells showed increased tumorigenicity compared with the vector-transfected cells. As expression of miR-17 decreased DU145 cell death, we examined tumor sections stained with H&E and detected extensive cell death in the control group (stained as red, arrows) relative to the miR-17 group (Figure 3b). The sections were then subject to TUNEL staining to detect apoptotic cells. There were many more apoptotic cells in the control group than in the miR-17 group (Figure 3c).

**Figure 4.**
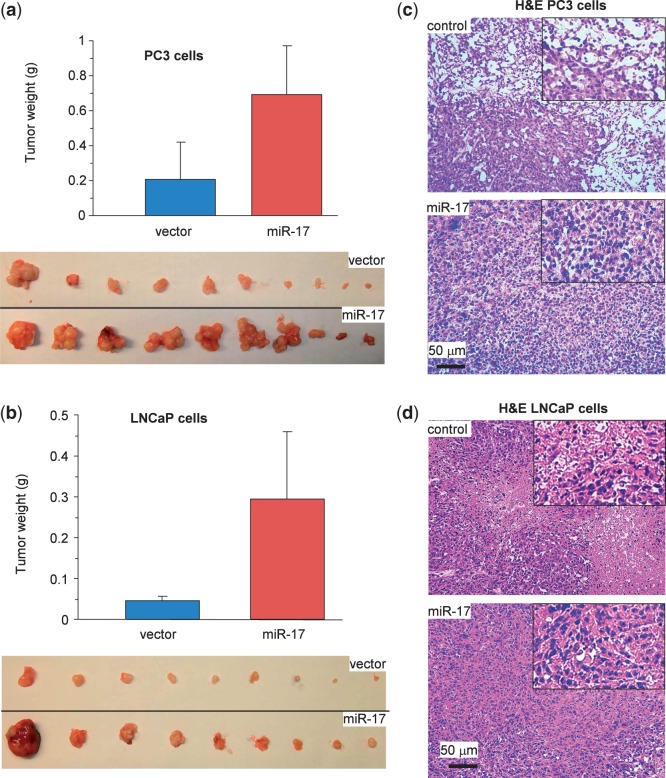
miR-17 enhances tumor growth in PC3 and LNCaP cells. (**a** and **b**) Tumor growth assay in PC3 [(a) 1 × 10^6^ cells/mouse for 4 weeks] and LNCaP [(b) 2.5 × 10^6^ cells/mouse for 6 weeks] cells transfected with miR-17 or the control vector. Expression of miR-17 increased tumor growth. Typical sizes of tumors are shown (lower). (**c** and **d**) H&E stained tumor sections were examined under a light microscope. Evidence of extensive cell death (decreased cell population) could be seen in the control but not in the miR-17 tumors.

We also conducted tumor formation assays in PC3 cells (Figure 4a) and LNCaP cells (Figure 4b). Expression of miR-17 increased tumor volume. Histological analysis of tumor sections showed that expression of miR-17 decreased tumor cell death in both PC3 cells (Figure 4c) and LNCaP cells (Figure 4d).

**Figure 5.**
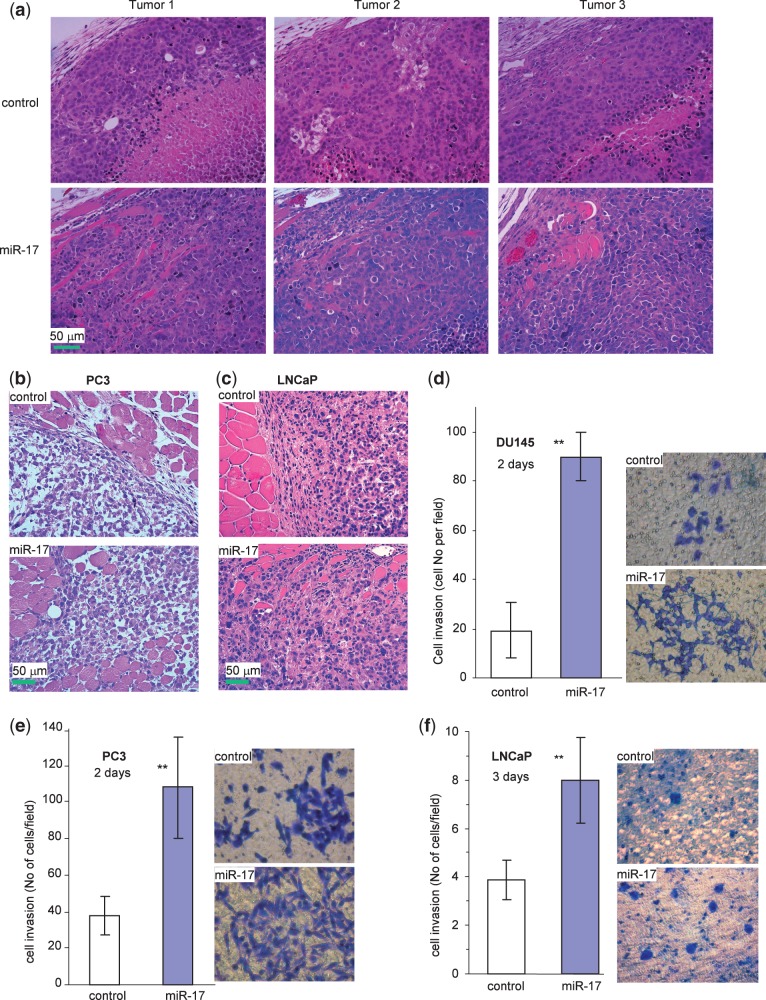
miR-17 affects tumor cell invasion. (**a**) The H&E stained tumor sections were examined under a light microscope. Evidence of invasive tissues could be seen in the miR-17 DU145 tumors, but it was absent in the control tumors. (**b** and **c**) Evidence of invasive tissues could also be seen in the miR-17 PC3 tumors (b) and miR-17 LNCaP tumors (c), but not in the control tumors. (**d–f**) Cell invasion was assayed in miR-17- and vector-transfected DU145 (d), PC3 (e) and LNCaP (f) cells using Matrigel-coated transwell chambers. The miR-17 cells were significantly more invasive than the control cells (left, **P < 0.01, *n* = 5). Typical photos are shown (right).

Interestingly, when we examined tumor sections in greater detail, we found that along the tumor boundaries, local invasion into the surrounding smooth muscle tissues was detected. This occurred in the miR-17 group but not in the control group (Figure 5a). Analysis of tumors formed by the PC3 (Figure 5b) and LNCaP cells (Figure 5c) also revealed that tumors formed by miR-17-transfected cells invaded the surrounding stroma at a higher rate as compared with the controls. To understand how expression of miR-17 might have induced tumor cell invasion, we conducted cell invasion assays in the DU145 (Figure 5d), PC3 (Figure 5e) and LNCaP (Figure 5f) cells transfected with miR-17 and the control vector placed in Matrigel-coated transwell chambers. We found that expression of miR-17 significantly enhanced cell invasion through Matrigel.

### Both miR-17-5p and miR-17-3p directly target TIMP3

**Figure 6.**
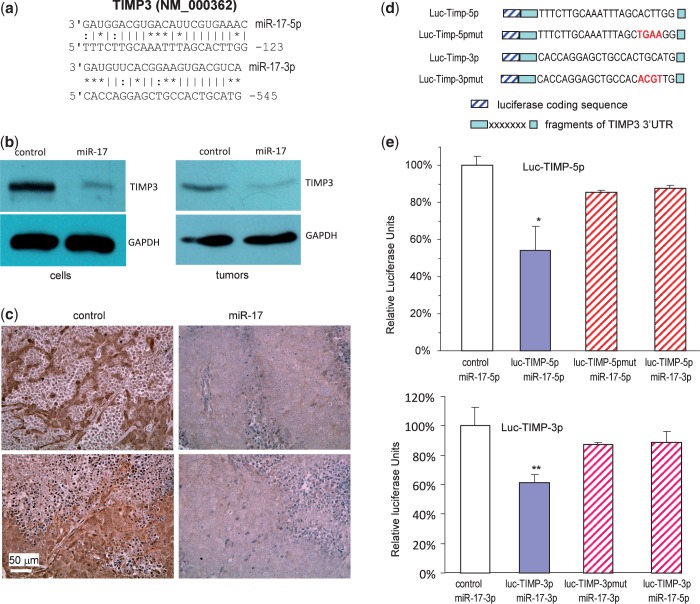
Both miR-17-5p and miR-17-3p represses TIMP3 expression. (**a**) Computational analysis indicated that miR-17-5p potentially targeted TIMP3 located at nucleotides 101–123 of the 3′UTR, while miR-17-3p potentially targeted TIMP3 located at 524–545 nt. (**b**) Left, cell lysate prepared from miR-17 or control-transfected cells was analyzed on western blot for TIMP3 expression. Repression of TIMP3 expression was seen in cells transfected with miR-17. Staining for GAPDH from the same membrane confirmed equal loading. Right, lysate prepared from tumors formed by the miR-17 or control-transfected cells was also analyzed for TIMP3 expression. Decreased TIMP3 level was seen in the miR-17 tumors compared with the control. (**c**) Sections from the miR-17 and control tumors were probed with anti-TIMP3 antibody. In general, tumor sections from the miR-17 transfected cells exhibited much lower levels of TIMP3 than the control. (**d**) Two luciferase constructs were generated, each containing a fragment harboring the target site of miR-17-5p and miR-17-3p, producing luc-TIMP-5p and luc-TIMP-3p. Mutations were also generated on the potential target sequence (red color), resulting in two mutant constructs luc-TIMP-5pmut and luc-TIMP-3pmut. (**e**) Upper, DU145 cells were co-transfected with miR-17-5p (or miR-17-3p) mimic and a luciferase reporter construct, which was engineered with a fragment of the TIMP3 3′UTR harboring miR-17-5p target site (luc-TIMP-5p) or a mutant (luc-TIMP-5pmut). An unrelated sequence was used as a control. The activity of luc-TIMP-5p decreased when co-transfected with miR-17-5p. Mutation of the binding site reversed miR-17-5p’s effect. Asterisks indicate significant differences. *P < 0.05, *n* = 6. Lower, DU145 cells were co-transfected with miR-17-3p (or miR-17-5p) mimic and luc-TIMP-3p or the mutant luc-TIMP-3pmut. Co-transfection with miR-17-3p mimic decreased luc-TIMP-3 activity but had no effect on the mutant.

In this study, we showed that the levels of both miR-17-5p and miR-17-3p were elevated in the miR-17 transfected cells compared with the control cells. We sought to identify the target(s) of both miR-17-5p and miR-17-3p in mediating the observed effects, focusing on proteins that were computationally predicted to be bound by miR-17-5p and miR-17-3p. Bioinformatics analysis indicated that there was one potential binding sites for miR-17-5p and one for miR-17-3p in the 3′UTR of TIMP3 (Figure 6a).

To test whether TIMP3 was repressed by miR-17 expression, we analyzed cell lysates prepared from the miR-17- and vector-transfected cells on western blot probed with anti-TIMP3 antibody and confirmed repression of TIMP3 in the miR-17-transfected cells as compared with the control (Figure 6b, left). To validate the targeting results, we analyzed TIMP3 expression in the tumors formed by cells transfected with miR-17 or the control vector. Western blot analysis indicated that TIMP3 level was much lower in the miR-17 tumors than in the control tumors (Figure 4b, right). Immunohistochemical results confirmed repression of TIMP3 levels in the miR-17 tumors compared with the control tumors (Figure 6c).

To obtain direct evidence that TIMP3 was a target of both miR-17-5p and miR-17-3p, we generated two TIMP3 luciferase reporter constructs (luc-TIMP-5p and luc-TIMP-3p), one harboring the binding site for miR-17-5p and the other for miR-17-3p, and two mutant constructs in which the binding sites were mutated (Figure 6d, Supplementary Figure S1 for detail information of the constructs). DU145 cells were co-transfected with miR-17-5p and luc-TIMP-5p or luc-TIMP-5pmut. Luciferase activity was significantly repressed when luc-TIMP-5p was co-transfected with miR-17-5p, but not with miR-17-3p. Mutation of the miR-17-5p target site abolished the inhibitory effects of miR-17-5p (Figure 6e). Similarly, when the cells were co-transfected with luc-TIMP-3p and miR-17-3p, but not miR-17-5p, we detected a significant inhibitory effect of miR-17-3p on luc-TIMP-3p activity, which was abolished when the target site was mutated (Figure 6e).

**Figure 7.**
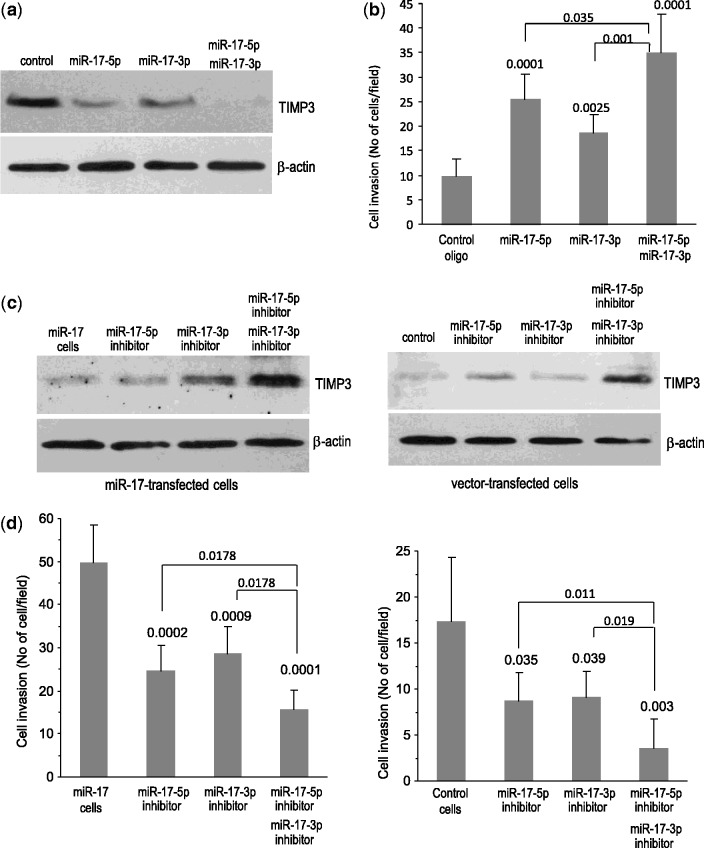
Confirmation of miR-17-5p and miR-17-3p targeting TIMP3. (**a**) DU145 cells transfected with RNA mimics (both miR-17-5p and/or miR-17-3p) or a control oligo were subjected to western blot analysis. Cells transfected with RNA mimics (both miR-17-5p and/or miR-17-3p) repressed TIMP3 expression. (**b**) In invasion analysis, cells transfected with both miR-17-5p and/or miR-17-3p RNA mimics promoted cell invasion. (**c**) The miR-17-transfected (left) and vector-transfected (right) DU145 cells were transiently transfected with RNA inhibitors (both miR-17-5p inhibitor and/or miR-17-3p inhibitor) or a control oligo. Cell lysates were subject to western blot analysis. Cells transfected with RNA inhibitor promoted TIMP3 expression. (**d**) The cells were also subject to invasion analysis, both types of cells transfected with miR-17-5p and/or miR-17-3p RNA inhibitor inhibited invasion.

To confirm the targeting of TIMP3 by both miR-17-5p and miR-17-3p, we transiently transfected DU145 cells with miR-17-5p and/or miR-17-3p RNA mimics. It was found that DU145 cells transfected with miR-17-5p mimics or miR-17-3p mimics significantly decreased the expression of TIMP3 protein levels (Figure 7a). When the cells were transfected with a combination of miR-17-5p and miR-17-3p, repression of TIMP3 was further increased. We then examined the effects of TIMP3 repression on cell invasion and found that inhibition of TIMP3 expression by the miR-17-5p mimic and the miR-17-3p mimic significantly promoted cell invasion (Figure 7b, Supplementary Figure S2a). As well, a combination of miR-17-5p and miR-17-3p exerted a greater effect on cell invasion than either one could when transfected alone.

To further confirm the targeting, we silenced ectopic expressed miR-17-5p and miR-17-3p or endogenous miR-17-5p and miR-17-3p. DU145 cells transfected with miR-17 were transiently transfected with a miR-17-5p inhibitor and/or a miR-17-3p inhibitor. Expression of TIMP3 was promoted by transfection of either miR-17-5p inhibitor or miR-17-3p inhibitor (Figure 7c, left). A combination of both inhibitors displayed greater promotion on TIMP3 expression than either one alone. Similarly, when the vector-transfected cells were transfected with either a miR-17-5p inhibitor or miR-17-3p inhibitor, expression of TIMP3 was enhanced (Figure 7c, right). Combining both inhibitors exerted a greater effect on enhancing TIMP3 expression, confirming a function of endogenous miR-17-5p and miR-17-3p. The effect of increasing TIMP3 expression was examined. We found that the promotion of TIMP3 expression by the inhibitors of miR-17-5p and miR-17-3p decreased the capacity of invasion in both miR-17-transfected cells (Figure 7d, left) and the vector-transfected cells (Figure 7d, right, Supplementary Figure S2b).

### Confirmation of TIMP3 in mediating miR-17 functions

**Figure 8.**
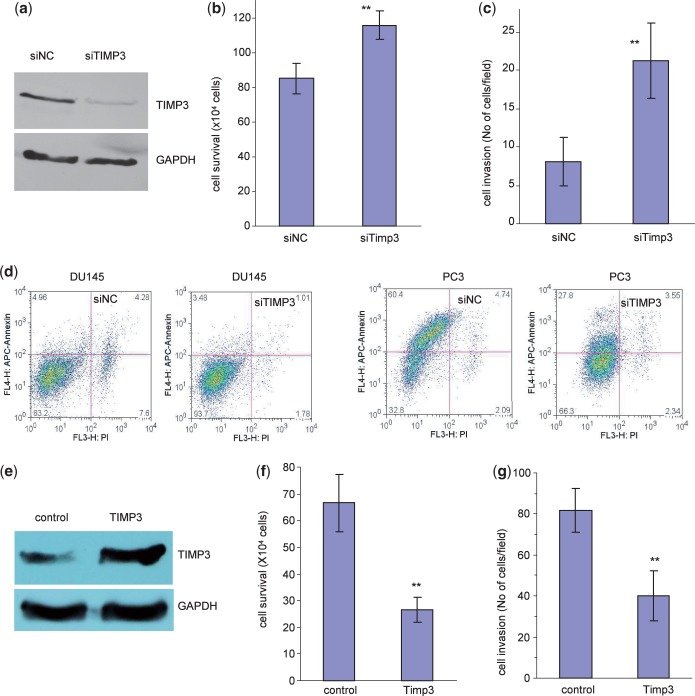
Confirmation of TIMP3 in mediating miR-17 functions. (**a**) Cell lysate prepared from DU145 cells transiently transfected with a control oligo or siRNA oligo targeting TIMP3 was subject to western blot analysis probed with anti-TIMP3 antibody to confirm silencing of TIMP3 expression by siRNA transfection. (**b**) DU145 cells transiently transfected with the TIMP3 siRNA or the control oligo were grown on 12-well tissue culture dishes in serum-free conditions for survival assay. Transfection with siRNA enhanced cell survival. **P < 0.011, *n* = 5. (**c**) DU145 cells transiently transfected with the TIMP3 siRNA or the control oligo were placed on matrigel coated transwell membranes for invasion assay. Transfection with siRNA enhanced cell invasion. **P < 0.001, *n* = 5. (**d**) DU145 and PC3 cells transiently transfected with the TIMP3 siRNA or the control oligo were examined for cell apoptosis. Transfection with siTIMP3 enhanced apoptosis of both types of cells. **P < 0.001, *n* = 3. (**e**) Cell lysates prepared from DU145 cells stably transfected with miR-17 and transiently transfected with TIMP3 expression construct or a control vector were subject to western blot analysis probed with anti-TIMP3 antibody. TIMP3 expression was increased by TIMP3 transfection. (**f**) The cells were subjected to cell survival assays. Transfection with TIMP3 reversed the effect of miR-17 resulting in decreased cell invasion. **P < 0.01, *n* = 10. (**g**) The cells were also subjected to cell invasion assays. Transfection with TIMP3 reversed the effect of miR-17 resulting in decreased cell invasion. **P < 0.01, *n* = 5.

To confirm the role of TIMP3 in mediating miR-17 functions, we delivered siRNA targeting TIMP3 into DU145 cells. Silencing of TIMP3 was confirmed by western blot (Figure 8a). Cell survival assays showed that transfection with TIMP3 siRNA significantly reduced cell death (Figure 8b, Supplementary Figure S3a). Examination of cell invasion showed that the TIMP3 siRNA-transfected cells exhibited increased capacity in cell invasion (Figure 8c, Supplementary Figure S3b). Cell apoptosis analysis indicated that transfection with the TIMP3 siRNA decreased apoptosis in DU145 and PC3 cells (Figure 8d). These results suggest that TIMP3 was involved in a pathway essential for miR-17-enhanced cell invasion and survival.

To finalize the function of miR-17 targeting TIMP3 in cell invasion, rescue experiments were performed. A TIMP3 expression construct was generated. DU145 cells stably transfected with miR-17 were transiently transfected with the TIMP3 expression construct or a control vector. There was an increase in TIMP3 protein expression when the rescue plasmid was transfected (Figure 8e). When the cells were grown in serum-free medium, the reintroduction of TIMP3 into the miR-17-expressing cells reversed the effect of miR-17 on cell survival: re-expression of TIMP3 was sufficient to cause cell death (Figure 8f, Supplementary Figure S3c). In cell invasion assay, when the TIMP3 overexpression construct was expressed, there was a decrease in cell invasion (Figure 8g, Supplementary Figure S3d). These results suggest that the effect of miR-17 on enhanced invasion and survival was at least partly occurring through repression of TIMP3 expression.

**Figure 9.**
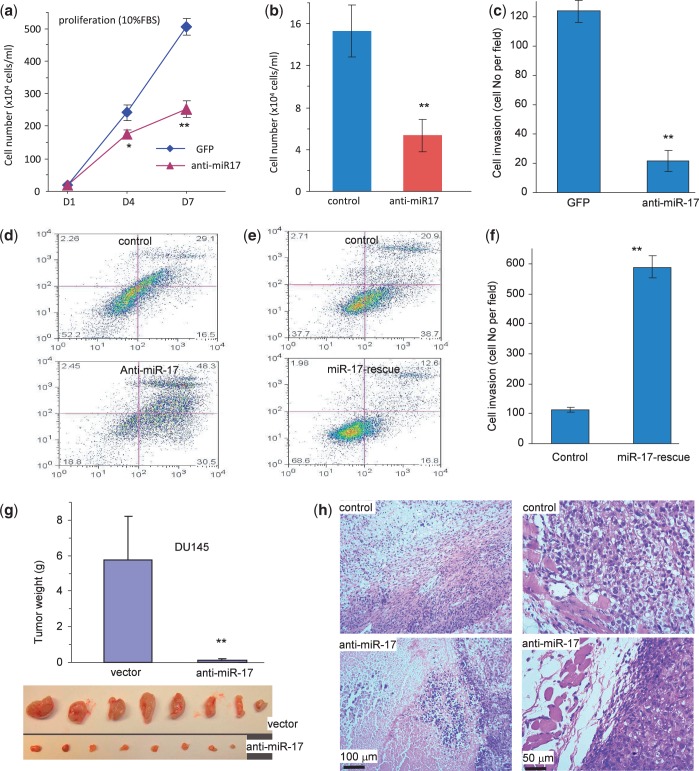
Confirmation of miR-17 functions. (**a–d**) DU145 cells transfected with the anti-miR-17 construct or a control vector were subject to proliferation, survival, apoptosis and invasion assays. Transfection with mR-17 reduced cell proliferation (a), survival (b) and invasion (c), but increased cell apoptosis (d). (**e** and **f**) The anti-miR-17-transfected cells were transiently transfected with a miR-17 mimic or a control oligo. Transfection with miR-17 rescued anti-miR-17’s effects on cell apoptosis (e) and invasion (f). (**g**) The anti-miR-17- and vector-transfected cells (1 × 10^6^ cells/mouse) were injected into nude mice, and the mice were maintained for 8 weeks. Expression of anti-miR-17 inhibited tumor growth. (**h**) H&E staining of tumor sections showed that there was increased cell death in the anti-miR-17 tumors as compared with the control (left). Although no sign of tumor invasion was seen, the anti-miR-17 tumor did not interact with the muscle tissue.

To test the functions of endogenous miR-17, we stably transfected DU145 cells with an anti-miR-17 construct or a control vector, as DU145 were found to express high endogenous levels of miR-17 (Figure 1e). In functional assays, we found that transfection with anti-miR-17 decreased cell proliferation (Figure 9a), survival (Figure 9b, Supplementary Figure S4a), invasion (Figure 9c, Supplementary Figure S4b), but increased cell apoptosis (Figure 9d). To confirm these results, we reintroduced a miR-17 mimic into the anti-miR-17-transfected cells. This was followed by an analysis of cell apoptosis and invasion, two assays that did not require long-term functionality of the transfected miR-17 mimic. These tests revealed a rescue effect of the miR-17 mimic on cell apoptosis (Figure 9e) and invasion (Figure 9f, Supplementary Figure S4c). Anti-miR-17-transfected cells were also subject to a tumor formation assay. We found that cells transfected with the anti-miR-17 construct formed smaller tumors as compared with the control (Figure 9g). Extensive cell death was detected in the smaller anti-miR-17 tumors, which displayed linking capacity to the muscle tissue (Figure 9h).

**Figure 10.**
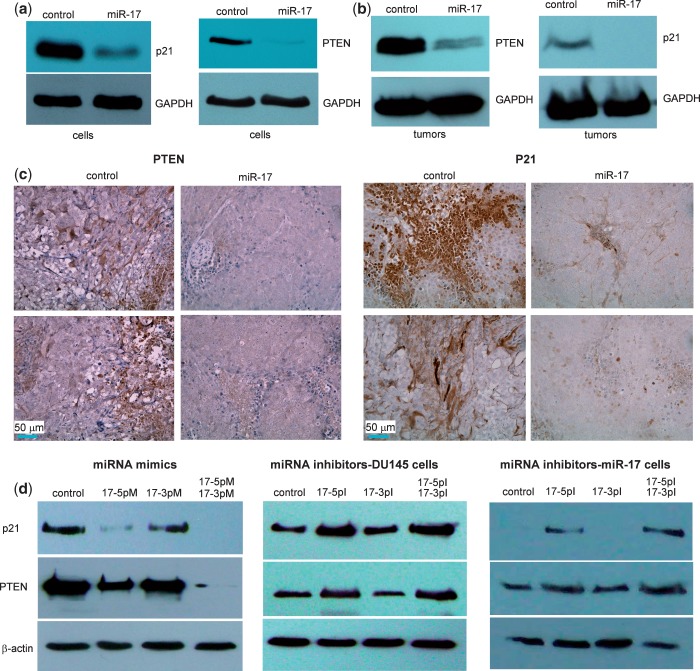
miR-17 represses TIMP3, PTEN and p21 expression in tumor sections. (**a**) Cell lysate prepared from miR-17 or control-transfected cells was analyzed on western blot for p21 and PTEN expression. Repression of p21 and PTEN expression was seen in cells transfected with miR-17. Staining for GAPDH from the same membrane confirmed equal loading. (**b**) Lysate prepared from tumors formed by the miR-17 or control-transfected cells was also analyzed for p21 and PTEN expression. Decreased p21 and PTEN levels were seen in the miR-17 tumors compared with the control. (**c**) Sections from the miR-17 and control tumors were probed with anti-PTEN and anti-p21 antibodies. In general, tumor sections from the miR-17 transfected cells exhibited much lower levels of PTEN and p21 than the control. (**d**) Individual and combined effects of miR-17-5p mimic (17-5pM) and miR-17-3p mimic (17-3pM) on p21 and PTEN expression was tested. 17-3pM minimally repressed p21 and PTEN expression, but 17-5pM showed a clear effect in repressing p21 and PTEN expression (left). DU145 cells transfected with a control (middle) or miR-17 vector (right) were treated with miRNA inhibitors. The miR-17-5p inhibitor (17-5pI) but not 17-3pI enhanced p21 and PTEN expression.

It had been previously reported that PTEN was targeted by miR-17 (30). We tested whether PTEN was a target in the cells used in our study. Cell lysates were prepared from DU145 cells stably transfected with miR-17 or the control vector and analyzed by western blotting probed with anti-PTEN antibody. We detected repression of PTEN expression in the miR-17-transfected cells as compared with the controls (Figure 10a). It had also been reported that p21 was a potential target of miR-17 (31). We examined p21 levels and confirmed that p21 expression was repressed in the miR-17-transfected cells (Figure 10a). These results confirmed that our system was comparable with those reported earlier. To further validate our system, we analyzed PTEN and p21 expression in the tumors formed by cells transfected with miR-17 or the control vector. Western blot analysis indicated that PTEN and p21 levels were much lower in the miR-17 tumors than in the control tumors (Figure 10b). Tumor sections were probed with anti-PTEN and anti-p21 antibodies. A clear reduction in the levels of these proteins was observed in the miR-17 tumor sections as compared with the controls (Figure 10c). We also tested the individual and combinatorial effects of miR-17-5p and miR-17-3p mimics, as well as miR-17-5p and miR-17-3p inhibitors on p21 and PTEN expression. Although the miR-17-3p mimic (17-3pM) showed weak activity in repressing p21 and PTEN expression, the miR-17-5p mimic (17-5pM) showed a clear repressive effect on p21 and PTEN expression (Figure 10d, left). The combination of both miRNA mimics produced a greater effect, perhaps, due to secondary signaling, which remains to be explored. The miR-17- and vector-transfected cells were also treated with the miRNA inhibitors. Although the miR-17-3p inhibitor (17-3pI) did not show any effects, the miR-17-5p inhibitor (17-5pI) enhanced p21 and PTEN expression (Figure 10d, middle for vector cells and right for miR-17 cells).

## DISCUSSION

The ability of prostate cancer to invade and metastasize are highly significant and allow prostate cancer cells to migrate through the extracellular matrix (ECM), which is composed of a wide variety of proteoglycans, glycoproteins, proteins and hyaluronan. The ECM plays an integral role in determining the shapes and functions of the cells. It also makes up the basement membrane and forms a physical barrier to avoid tumor cell invasion and metastasis. Cancer cells are capable of degrading the ECM molecules and the physical barrier by producing the enzymes matrix metalloproteinases (MMPs), which are responsible for cancer cell invasion, tissue remodeling, wound healing, angiogenesis, metastasis and cancer progression (32,33). It has been reported that MMPs play crucial roles in the invasion and metastasis of prostate cancer (34). Normally, the activities of MMPs are inhibited by one or more of the four endogenous tissue inhibitors of metalloproteinases (TIMP1-4). However, in many types of cancers, especially in prostate cancer, MMP expression is upregulated, whereas TIMP expression is downregulated (35). One mechanism associated with TIMP downregulation is promoter hypermethylation. However, although immunohistochemical analysis showed decreased TIMP3 levels in high-grade primary prostate cancers, promoter hypermethylation was only detected in a small proportion of high-grade specimens (36). This suggested that other mechanisms may also play roles in the downregulation of TIMP expression. Herein we showed that repression of TIMP3 expression could be achieved by a miRNA miR-17.

Although abnormal miR-17 expression is frequently observed in various types of cancers (37–39), the role of miR-17 in regulation of prostate cancer remains unclear. Our study has demonstrated that miR-17 played an important role in the development and invasion of prostate cancer. As TIMP3 is a tumor suppressor frequently found downregulated in prostate cancer, our work is of clinical relevance, which may lead to a new approach in interrupting prostate cancer development and invasion by targeting miR-17 expression.

It was previously reported that p21 and PTEN were targeted by miR-17 (40–42). One important function of miR-17 in tumor proliferation is the inhibition of p21, which is a target of miR-17-5p. It was shown that the miR-17-5p targets the E2F1 pathway and the cell-cycle inhibitor, p21/WAF1 (41,43). In this function, miR-17-5p promotes cell-cycle progression and hyperproliferation, which is consistent with our observation that miR-17 transfected prostate cancer cell lines tend to have increased proliferation rates. Another important function of miR-17-5p in tumor formation and development is the inhibition of PTEN, a well-characterized tumor suppressor. As a target of miR-17-5p, PTEN can regulate the PI3K/AKT pathway, a major cell survival pathway, which plays an important role in prostate cancer (44–46).

In this study, we also found that both p21 and PTEN were targets of miR-17 in prostate cancer cells and tumors. Our finding that expression of miR-17 enhanced cell survival correlated with p21 and PTEN downregulation was consistent with previously reported results. Thus, TIMP3, p21 and PTEN were collectively targeted by miR-17 in prostate cancer. As expression of some miRNAs in the miR-17∼92 cluster and its paralogs is upregulated in prostate cancer patients (47), miR-17 targeting TIMP3, p21 and PTEN appears to be a crucial event in prostate cancer development and invasion. This may explain why ectopic expression of miR-17 significantly promoted prostate tumor growth and invasion. In one of the extreme cases, we found that ectopic expression of miR-17 elevated tumor growth by >10-fold, as it repressed expression of these tumor suppressors, enhancing tumor cell proliferation, survival and invasion. Our study and others suggest that miR-17 functions as a powerful oncogenic miRNA in prostate cancer.

A miRNA precursor can be processed to produce two single-stranded miRNAs. Although it is well accepted that the guide strand, known as the miRNA, binds to an mRNA-induced silencing complex and produces its effect on the target genes (48). In contrast, the passenger strand, also known as miRNA* (miRNA star), is degraded and thought to have no functional relevance. Further studies have found that some miRNA precursors can be processed to produce abundant levels of both miRNA strands. Wang and colleagues have shown that miR-17-5p and miR-17-3p are differentially expressed during different time point in cell culture (49). We have also reported that both miR-17-5p and miR-17-3p are highly expressed in miR-17 transgenic mice (22). In this study, we reported that miR-17-5p and miR-17-3p were not only both abundantly expressed from one single precursor but also acted in coordination to enhance the power of a miRNA precursor by repressing the same target. Our results highlight the importance that both mature miR-17-5p and the passenger strand miR-17-3p targeted the same target TIMP3, which would enhance the power of a miRNA. A miRNA is known to play a role in fine-tuning protein synthesis. Although an mRNA may be regulated by multiple miRNAs, expression of these miRNAs in the regulatory network is complex: they may not be expressed at the levels sufficiently for repression of the mRNA. Targeting the same mRNA (e.g. TIMP3) by both strands of a miRNA (e.g. miR-17-5p and miR-17-3p) can effectively and efficiently exert the biological functions of a miRNA. Our study showed that ability of mature miR-17-5p and the passenger strand miR-17-3p to synergistically enhance prostate tumor growth and invasion by repressing the same target TIMP3 may be a mode of miRNA-mediated gene regulation.

## SUPPLEMENTARY DATA

Supplementary Data are available at NAR Online.

## FUNDING

Canadian Institutes of Health Research [MOP-102635, MOP-111171 to B.B.Y.], Career Investigator Award [CI 7418 to B.B.Y.] from the Heart and Stroke Foundation of Ontario. Funding for open access charge: Canadian Institutes of Health Research [MOP-102635, MOP-111171 to B.B.Y.].

*Conflict of interest statement*. None declared.

## Supplementary Material

Supplementary Data
