# Supplementary material for: Both mature miR-17-5p and passenger strand miR-17-3p target TIMP3 and induce prostate tumor growth and invasion
Source: Nucleic Acids Res. 2013 Aug 28;41(21):9688–704. doi: 10.1093/nar/gkt680 (PMC3834805; doi:10.1093/nar/gkt680)
Supplement: Supplementary Data [file supp_41_21_9688__index.html]

Both mature miR-17-5p and passenger strand miR-17-3p target TIMP3 and induce prostate tumor growth and invasion — Both mature miR-17-5p and passenger strand miR-17-3p target TIMP3 and induce prostate tumor growth and invasion — Supplementary Data 

# Both mature miR-17-5p and passenger strand miR-17-3p target TIMP3 and induce prostate tumor growth and invasion

## 

files

**Files in this Data Supplement:**

- Supplementary Data - pdf file
