# Supplementary material for: Both mature miR-17-5p and passenger strand miR-17-3p target TIMP3 and induce prostate tumor growth and invasion
Source: Nucleic Acids Res. 2013 Aug 28;41(21):9688–704. doi: 10.1093/nar/gkt680 (PMC3834805; doi:10.1093/nar/gkt680)
Supplement: Supplementary Data [file supp_gkt680_nar-01799-y-2013-File003.pdf]

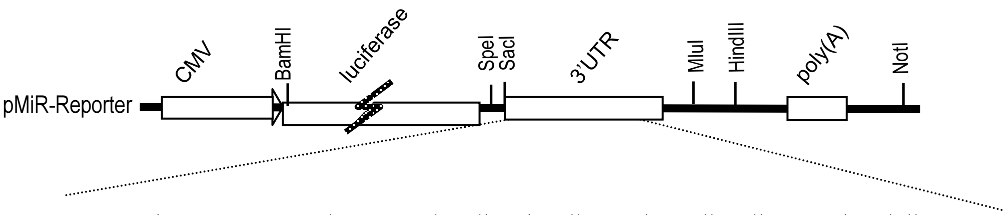

Luc-TIMP3^{-17-5p} (Luc-TIMP-5p)

GAGCTCtgagcgccagaccctgccccacctcacttccctcccttcccgctgagcttcccttggacactaactcttcccag
atgatgacaatgaaattagtgcctgt**tttcttgcaaatttagcacttgg**aacatttaaagaaaggtc ACGCGT

Luc-TIMP3-mut^{-17-5p} (Luc-TIMP-5pmut)

GAGCTCtgagcgccagaccctgccccacctcacttccctcccttcccgctgagcttcccttggacactaactcttcccag
atgatgacaatgaaattagtgcctgt**tttcttgcaaatttagctgaagg**aacatttaaagaaaggtc ACGCGT

Luc-TIMP3^{-17-3p} (Luc-TIMP-3p)

GAGCTCggaacctgtattcctcttcttcgtgataatataatctctatttttttaggaaaacaaaaatgaaaaact
actccatttgaggattgtaattcccacccctcttgcttcttccccacctcaccatctcccagaccctcttccctttgcc
cttctcctccaatacataaaggacacagacaaggaacttgctgaaaggccaaccatttcaggatcagtcaaa
ggcagcaagcagatagactcaaggtgtgtgaaagatgttata**caccaggagctgccactgcatg**tccca
accagactgtgtctgtc ACGCGT

Luc-TIMP3-mut^{-17-3p} (Luc-TIMP-3pmut)

GAGCTCggaacctgtattcctcttcttcgtgataatataatctctatttttttaggaaaacaaaaatgaaaaact
actccatttgaggattgtaattcccacccctcttgcttcttccccacctcaccatctcccagaccctcttccctttgcc
cttctcctccaatacataaaggacacagacaaggaacttgctgaaaggccaaccatttcaggatcagtcaaa
ggcagcaagcagatagactcaaggtgtgtgaaagatgttata**caccaggagctgccacacgttg**tccca
accagactgtgtctgtc ACGCGT

Fig S1. Sequences of luciferase constructs.

Fragments of TIMP3 3'UTRs were inserted into the luciferase report vector pMir-Report producing constructs Luc-TIMP3-R17-5p and Luc-TIMP3-R17-3p. The potential miR-17-5p and miR-17-3p target sites were labeled in blue. Mutations labeled in red were generated in the miR-17-5p and miR-17-3p target sites producing mutant constructs Luc-TIMP3-R17-5p-mut and Luc-TIMP3-R17-3p-mut.

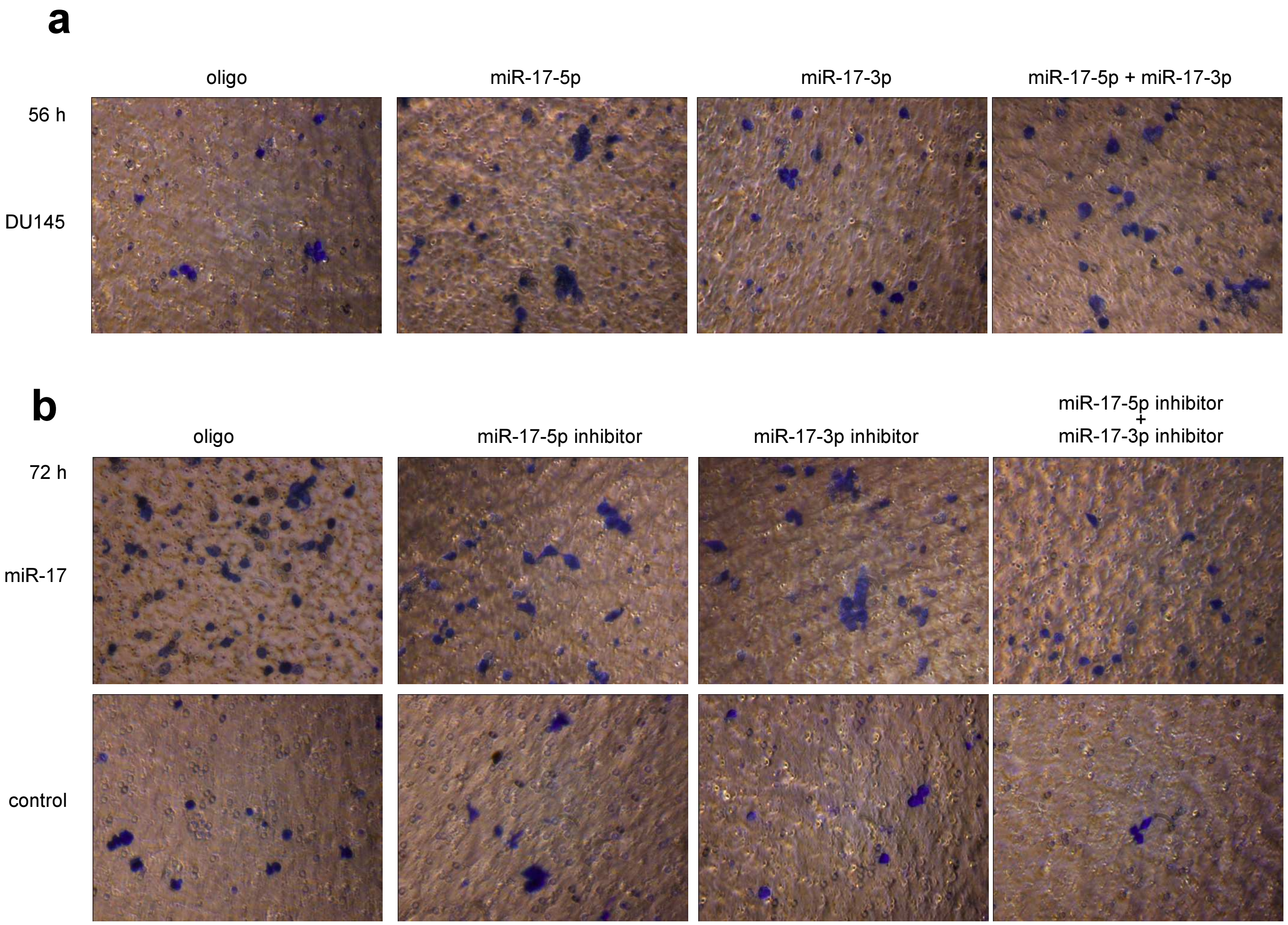

Fig S2. Confirmation of miR-17-5p and miR-17-3p targeting TIMP3.

(a) In invasion analysis, cells transfected with both miR-17-5p and/or miR-17-3p RNA mimics promoted cell invasion.

(b) The miR-17-transfected (left) and vector-transfected (right) DU145 cells were subject to invasion analysis, both types of cells transfected with miR-17-5p and/or miR-17-3p RNA inhibitor inhibited invasion.

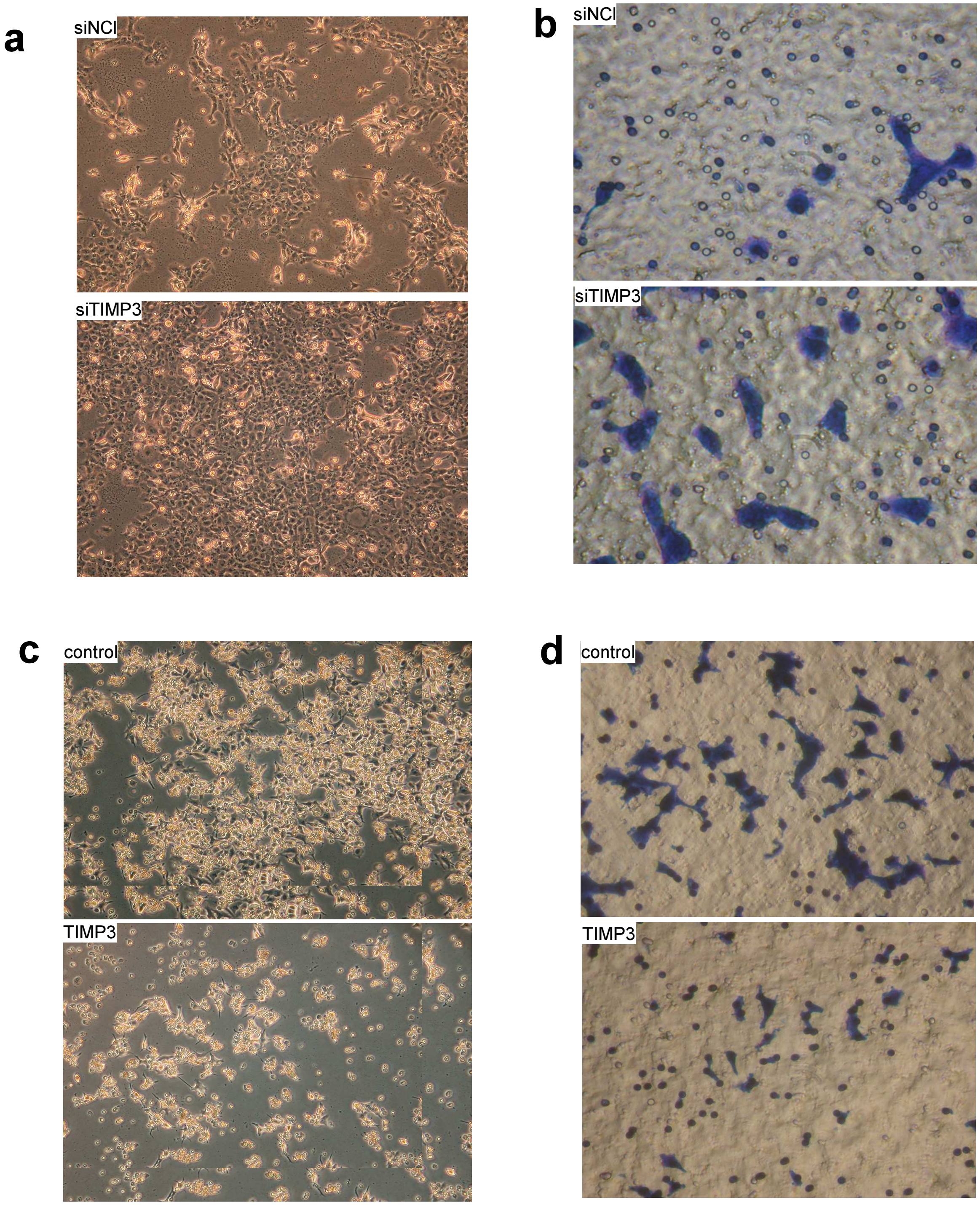

Fig S3. Confirmation of TIMP3 in mediating miR-17 functions.

(a) DU145 cells transiently transfected with the TIMP3 siRNA or the control oligo were grown on 12-well tissue culture dishes in serum-free conditions fro survival assay. Transfection with siRNA enhanced cell survival.

(b) The DU145 cells transfected with siRNA oligo targeting Timp3 or a control oligo were subjected to invasion assays for 48 hours. Transfection with siRNAs increased invasion.

(c) Ectopic expression of TIMP3 in the miR-17 cells reversed miR-17 effect on DU145 cell survival.

(d) The cells were also subjected to cell invasion assays. Transfection with TIMP3 reversed the effect of miR-17 resulting in decreased cell invasion.

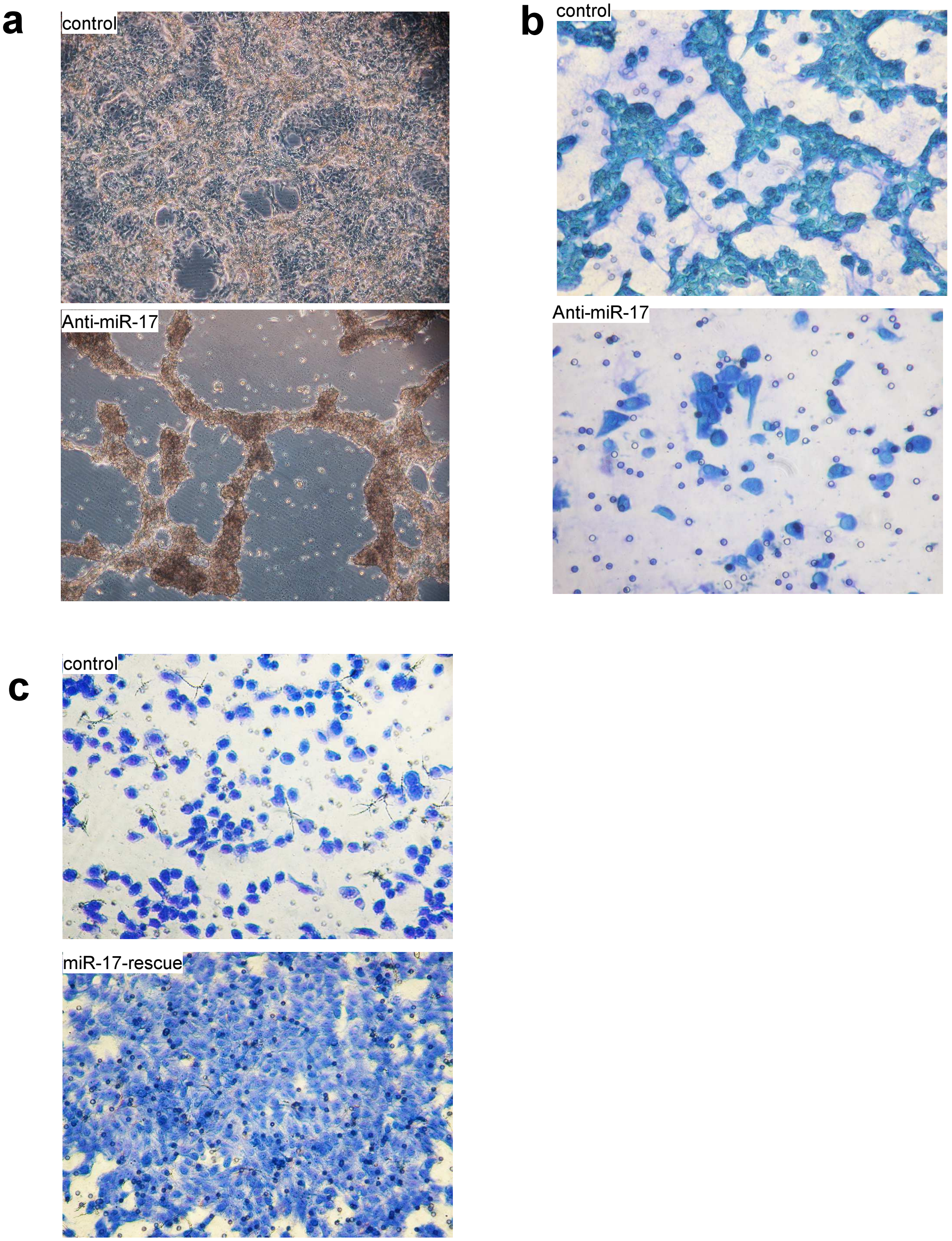

Fig S4. Confirmation of miR-17 functions.

(a-b) Cells transfected with the anti-miR-17 construct or a control vector were subject to assays for cell survival and invasion. Transfection with mR-17 reduced cell survival (a), and invasion (b).

(c) The anti-miR-17-transfected cells were transiently transfected with miR-17 mimic or a control oligo. Transfection with miR-17 rescued anti-miR-17's effects on cell invasion.
